# Exploring the knowledge and attitudes towards metabolic dysfunction associated fatty liver disease (MAFLD): Validation and correlations of MAFLD-knowledge questionnaire and MAFLD-attitude questionnaire

**DOI:** 10.1016/j.heliyon.2024.e40217

**Published:** 2024-11-07

**Authors:** Samah Al Tawil, Mohamad Abdelkhalik, Adam El Fouani, Nour Allakiss, Lama Mattar, Wissam H. Faour, Rajaa Chatila

**Affiliations:** a Gilbert and Rose-Marie Chagoury School of Medicine, Lebanese American University, Byblos, Lebanon; b Lebanese American University Medical Center – Rizk Hospital, Beirut, Lebanon; c Natural Sciences Department, School of Arts and Sciences, Lebanese American University, Byblos, Lebanon

## Abstract

**Background:**

Metabolic dysfunction associated fatty liver disease (MAFLD) is a highly prevalent non-communicable disease whose prevalence is reaching pandemic proportions. Its implications constitute a major public health concern. To date, no validated tool measures knowledge and attitudes towards MAFLD in young adults in the Middle East and North Africa region.

**Objective:**

To establish and validate questionnaires that measure knowledge and attitudes towards MAFLD.

**Methods:**

MAFLD-Knowledge Questionnaire (KQ) and MAFLD-Attitudes Questionnaire (AQ) were developed by disease content experts and piloted on a group of 20 students. The questionnaire was next administered to 406 university students aged 18–24 years. The dimensionality of the instrument was tested using exploratory factor analysis and consistency with Cronbach's alpha. Finally, known-group validity was assessed by comparing the knowledge and attitudes of those with poor versus good knowledge.

**Results:**

For the knowledge questionnaire, 28 out of 32 questions had good loading and were thus included. Based on Exploratory Factor Analysis (EFA), there were 4 domains with Kaiser–Meyer–Olkin (KMO) 0.95 and Bartlett test with P < 0.001, with very good internal consistency (Cronbach's alpha 0.88). For the attitudes questionnaire, 17 items were extracted all with adequate loading. EFA revealed 3 domains with KMO of 0.95 and very good internal consistency (Cronbach's alpha 0.81). Known group validity showed a significant difference between the attitudes of subjects with poor and moderate knowledge and poor and high knowledge scores but not between moderate and high knowledge scores.

**Conclusion:**

We developed and validated two questionnaires, one for the assessment of knowledge and the other for attitudes towards MAFLD in young adults. Further studies are needed to confirm dimensionality and reproducibility in different populations and age groups.

## 1 Introduction

Non-alcoholic fatty liver disease (NAFLD) is characterized by the presence of hepatic steatosis, in the absence of significant alcohol consumption or other underlying causes of steatosis such as medications. NAFLD is a broad term that encompasses a wide range of liver conditions, ranging from simple steatosis to non-alcoholic steatohepatitis (NASH), leading to more serious conditions such as fibrosis, cirrhosis, hepatocellular carcinoma (HCC) and end-stage liver disease requiring transplantation [1]. It has become a significant global health concern, affecting approximately 30 % of the world's population, according to recent data [2] with predictions to soon become the major indication for liver transplantation, surpassing hepatitis C [3,4]. However, ongoing efforts to refine the definition of this condition in the recent years have led to moving away from using the term “NAFLD”, especially that the term makes the diagnosis that of exclusion. Metabolic dysfunction associated fatty liver disease (MAFLD) was defined by Eslam et al., in 2020 as presence of fatty liver disease in addition to the presence of diabetes mellitus, being overweight or presence of metabolic dysfunction criteria without any requirement for exclusion of other factors and widely used in Asian-Pacific countries [5] Emanating from this new term, another term was introduced by Western countries, metabolic dysfunction-associated steatotic liver disease (MASLD), proposed as a more precise definition of the condition reflecting the metabolic roots of the disease and aligning with related concepts, such as metabolic dysfunction-associated steatohepatitis (MASH). It also introduces new definitions, such as metALD (metabolic-associated alcoholic liver disease), to provide a more comprehensive framework for understanding the spectrum of liver disease [6]. These new definitions result in subtle differences when applied to the same population and should not be confused [[7], [8], [9]]. Unfortunately, an ongoing debate about the adoption of the newer definition continues [[10], [11]], but in our work we chose to use the term MAFLD, knowing it has been endorsed by key healthcare authorities [12,13].

The prevalence of MAFLD has been found to be approximately 40 %, according to a recent meta-analysis [14] with some studies reporting even higher prevalence rates reaching up to 50 % of the population [15]. This highlights the widespread impact of MAFLD on global health. Moreover, MAFLD seems to represent a population with a severer metabolic burden compared to NAFLD [16,17]. In fact, MAFLD is an independent predictor of cardiovascular disease, which is the primary cause of mortality in patients with this disease [18]. It can lead to severe liver disease and associated complications including HCC which has a high mortality index [19]. The direct and indirect costs of MAFLD are high, with the annual healthcare expenditure in the US reported to be over $100 billion [20]. Not only Western countries, but the Middle East and North Africa (MENA) region is also known to have high prevalence rates of MAFLD of around 32 % [4]. Yet, despite this high prevalence and disease burden worldwide, a knowledge gap about the disease continues to exist [21]. The exact prevalence data about MAFLD in Lebanon is lacking, yet, data about the prevalence of metabolic syndrome in the general Lebanese population is believed to be high [22].

Taking that into consideration, understanding the awareness and attitudes of the public regarding this disease is important. Using and validating scales to assess the knowledge of and attitudes towards this disease will be useful to identify the gaps and decrease stigma [23]. Furthermore, assessing the general population's knowledge and attitudes regarding MAFLD is crucial to design appropriate public health interventions and enhance the care of this disease [24]. Moreover, increased knowledge can result in timely diagnosis and superior patient management [20]. It can also enhance the perceived severity and susceptibility of MAFLD and consequently prompt behavioral changes towards a healthier lifestyle [25]. Therefore, addressing awareness and understanding of MAFLD among young adults is pivotal, given the current lack of awareness in the face of the increasing prevalence of the disease as this could shape interventions aimed at preventing the progression towards advanced liver diseases and cirrhosis. Thus, further research and public health initiatives, especially among the youth, are required to manage the increasing prevalence of MAFLD and enhance disease prevention [26].

While some studies have validated awareness and knowledge questionnaires for MAFLD in the general adult population [[27], [28], [29]], there is a notable gap in validation efforts specific to young adults. This demographic group lacks sufficient validated questionnaires assessing their knowledge and attitudes towards MAFLD. For instance, Du et al. (2023) assessed and validated a self-administered questionnaire evaluating awareness and knowledge of MAFLD among Chinese young adults aged 18–25 years [30]. However, this study didn't validate the questionnaire regarding attitudes towards the disease. Knowing that attitudes inform about disease prevention, outcome and the setting of policies as shown in numerous research papers on both communicable and non-communicable diseases [[31], [32], [33]], it would be necessary to develop a new tool for evaluating disease knowledge and as well as attitudes of young adults towards MAFLD.

To fill this gap, we designed two questionnaires: The Metabolic Dysfunction-associated Fatty Liver Disease Knowledge Questionnaire (MAFLD-KQ) and the Metabolic Dysfunction-associated Fatty Liver Disease Attitude Questionnaire (MAFLD-AQ) and we hypothesized that a standard validation might demonstrate the validity and reliability of these newly designed questionnaires to assess the knowledge and attitudes of young adults regarding MAFLD. In this study, we describe the phases of MAFLD-KQ and MAFLD-AQ development and the process of their validation, including content validity, internal consistency, construct validity, and known group validity.

## 2 Methods

### 2.1 Questionnaire development and content validity

The development and analysis of the questionnaire were carried out in two main phases. Initially, the questionnaire was created after thoroughly reviewing existing literature on knowledge and attitudes about MAFLD. This involved identifying relevant questions and scales used in previous surveys related to MAFLD. In the first phase, experts including a medical doctor, a biostatistician, epidemiologist, and a pharmacologist assessed the draft questionnaire to ensure its content accurately represented the intended concepts and theories. The second phase involved psychometric testing using Exploratory Factor Analysis (EFA) to evaluate the questionnaire's validity and reliability. The knowledge section was developed based on the etiology, transmission, risk, complications and symptoms of MAFLD while the attitude section of the questionnaire was developed based on different concepts and theories obtained in previous studies [34,35]. The final questionnaire was divided into different sections including demographics, medical information, familial history of diseases associated with NAFLD, and both scores of Knowledge and Attitudes. An assessment of the knowledge and attitudes of the participants about the disease and an evaluation of their risk profile in relation with the disease was previously completed and the findings are now available for more reference [36].

The initial version of the questionnaire formulated for this study was written in English to ensure consistency and accuracy among the questions. To test whether the questionnaire seemed appropriate and understandable, face validity checks were performed with 20 participants. This process evaluated how well participants understood the questions and how relevant the questions appeared to them.

### 2.2 Study instrument

The questionnaire consisted of both open-ended and closed-ended questions and was well received by participants. There were three sections in the final questionnaire: (1) socio-demographic characteristics of participants; (2) knowledge of MAFLD; and (3) attitudes towards MAFLD. Sociodemographic characteristics studied included age, gender, height and weight, marital status, level of education, smoking history, physical activity presence or family history of comorbidities.

To assess disease knowledge, a MAFLD-KQ scale was developed, by adapting a questionnaire consisting of twenty-seven questions from a previous study conducted by Chen et al., in 2019. Our research team supplemented this scale with two additional questions on risk factors and three questions regarding disease progression, lifespan comparison, and the impact of early diagnosis and treatment. Questions on various aspects of MAFLD were awarded one point when answered correctly. These included prevalence, reversibility, health consequences, symptoms, diagnosis, treatment, and risk factors. Questions were in true-false and multiple-choice format. The responses of the participants to individual questions of the knowledge score were summed up by adding the individual scores of all the items.

To assess the attitudes regarding MAFLD, a 17-question scale (MAFLD-AQ) was developed. The questions were in Likert scale format (1–5) and the total score was conducted by summing up responses to individual questions, giving a final score between 17 and 85. The content of the survey was checked for accuracy by all authors.

### 2.3 Study design and sample size

A cross-sectional study was conducted between December 2023 and January 2024 using a snowball sampling of different university students from five different districts of Lebanon. Younger population such as University students were chosen because of their exposure to different sources of medical information such as medical websites and social media. Moreover, young adults might have different risk factors, lifestyle habits, or awareness levels compared to other age groups. Validation helps tailor the questionnaire to the specific context of this demographic, ensuring that questions are relevant and appropriately phrased for this age group. Since a minimum of 5–10 participants per scale item would be adequate for establishing sufficient evidence of scale validity and reliability [37], and having a knowledge and attitudes scores comprised of 32 and 17 items respectively (total of 49 items), the minimum target sample size for validation was set to be 250 participants. A final number of 406 participants was included to account for missing values. All participants older than 18 years old, without any cognitive or intellectual problems, were invited to fill in the questionnaire. The target population comprised university students aged 18–26 years enrolled in various Lebanese universities across Lebanon. Exclusion criteria included populations outside the specified age range.

### 2.4 Study conduct and measurements

A team of researchers specializing in gastrointestinal and liver diseases, particularly MAFLD, conducted the data collection for the study. Participants either filled out the questionnaires on their own or were interviewed, with each questionnaire taking about 10–15 min to complete. Sampling was conducted through online platforms including Instagram, Facebook, Twitter, LinkedIn, and WhatsApp. Participants who provided informed consent were included in the study. Initially, participants were briefed about the study's purpose and details, after which informed consent was obtained from those who agreed to participate.

### 2.5 Ethical statement

The study was approved by the Lebanese American University (LAU) Institutional Review Board (LAUIRB) under the approval number: IRB #: LAU.SOM.RC1.19/Dec/2023. All participants provided written consent before they could participate. Participation was voluntary and participants could opt out at any time if they felt uncomfortable. All collected data had no personal identifiers, was kept confidential and was accessible only to the research group.

### 2.6 Statistical analysis

Statistical analyses were performed using Statistical Package for Social Science (SPSS) version 23 (IBM SPSS Software, Chicago, IL, USA). Descriptive statistics were calculated using mean and standard deviation for continuous measures, counts and percentages for categorical variables.

### 2.7 Assessment of the internal consistency of the MAFLD-KQ and MAFLD-AQ

Cronbach's alpha coefficients were computed to characterize the internal consistency reliability of the Arabic UAS7 and CU-Q2oL. Its interpretation was defined as follows: A coefficient <0.6 was considered unacceptable, 0.60–0.64 as undesirable, 0.65–0.69 as minimally acceptable, 0.70–0.79 as respectable, 0.8–0.90 as excellent, and >0.9 as excessive consistency [38].

### 2.8 Assessment of the content validity of the MAFLD-KQ and MAFLD-AQ

To confirm the content validity of both knowledge and attitudes scores, an EFA was launched using Promax rotation, since the extracted factors were found to be significantly correlated. The retained number of factors corresponded to Eigenvalues higher than one. The Kaiser–Meyer–Olkin (KMO) measure of sampling adequacy and Bartlett's test of sphericity were ensured to be adequate. The KMO measures sampling adequacy for each variable in the model and for the complete model, whereas Bartlett's test would indicate if the variables were unrelated and therefore unsuitable for structure detection.

### 2.9 Assessment of the construct validity of the MAFLD-KQ and MAFLD-AQ

Construct validity indicates the correlation with other standard measurements. Spearman and Pearson's correlation coefficients of <0.3, 0.3–0.6, and >0.6 were considered to indicate weak, moderate, and strong correlations respectively [39]. Known-group validity demonstrates the ability to discriminate between groups that are assumed to be different. Known-group validity and the ability of MAFLD-AQ to distinguish patients with three knowledge levels were investigated using the Kruskal-Wallis test.

## 3 Results

### 3.1 Socio-demographic characteristics

406 individuals participated in this study. Gender distribution was even between males and females, comprising 50.7 % and 49.3 %, respectively. The average age and BMI were 22.3 ± 2.32 and 26.06 ± 4.44, respectively. The majority of participants were single (91.1 %), belonged to middle socioeconomic status, and were not healthcare professionals (62.6 %). Hyperlipidemia was the most prevalent preexisting metabolic abnormality among participants (34.5 %), while hypertension topped the list among family members (60.3 %). 44.1 % of the participants perceived themselves as being physically inactive and less than a quarter of the participants exercise more than 4 times a week. Further sociodemographic and medical information can be found in Table 1.

**Table 1:** Sociodemographic characteristics of study participants.

Characteristics	n (406)	Percentage (%)
**Gender**		
Males	206	50.7
Females	200	49.3
**Are you specialized in healthcare?**		
No	254	62.6
Yes	152	37.4
**Social status**		
Single	370	91.1
Married	32	7.9
Divorced/Separated	4	1.0
**Economic Status**		
Low	41	10.1
Middle	206	50.7
High	159	39.2
**Smoking Status**		
I have never smoked	131	32.3
Occasional Smoker	29	7.1
Current cigarette/e-cigarette smoker	186	45.8
Current nargileh smoker	49	12.1
**Medical History**		
Diabetes Mellitus	142	35.0
Hypertension	133	32.8
Hyperlipidemia	140	34.5
Heart Disease	126	31.0
MAFLD	105	25.9
**Family History**		
Diabetes Mellitus	232	57.1
Hypertension	245	60.3
Hyperlipidemia	241	59.4
Heart Disease	179	44.1
MAFLD	112	27.6
**Physical activity**		
Not active	179	44.1
Once or twice per week	125	30.8
3–4 times per week	70	17.2
More than 4 times per week	32	7.9
	**Mean**	**SD**
**Age**	22.3	2.32
**BMI**	26.06	4.44

Notes. Metabolic dysfunction-associated fatty liver disease (MAFLD), Body Mass Index (BMI).

### 3.2 Exploratory factor analysis (EFA)

#### 3.2.1 Exploratory factor analysis for MAFLD-KQ

Out of the 32 items of the knowledge score, item 1 (MAFLD disease is common among the general population) was removed because it was not significantly associated with other different items of the knowledge questionnaire. Items 6 (there are no adverse consequences for MAFLD), 21 (drinking too much alcohol can cause MAFLD) and 28 (some medications or natural supplements for weight loss may reverse disease progression) were also removed because of their low communalities (<0.3). All other 28 items could be extracted from the list since none of them was over-correlated to the other (r > 0.8) or had a low loading on factors (<0.3). The EFA was run over the whole sample population (n = 406) and demonstrated a four-domain structure which described 50.15 % of the variance of the 28 remaining items. The KMO measure of sampling adequacy was found to be 0.95, with a significant Bartlett's test of sphericity (p < 0.001), indicating the adequacy of the factor analysis (Table 2).

**Table 2:** Direct Promax exploratory factor analysis of the MAFLD-KQ.

	Extraction of Sums of Squared Loadings				Rotation Sums of Squared Loadings
Domain	Total	% of Variance	Cumulative %		Total
**I**	11.804	42.158	42.158		7.936
**II**	1.196	4.27	46.428		2.821
**III**	0.613	2.189	48.617		1.752
**IV**	0.524	1.87	50.487		1.627

		**Domains**			
**Item No.**	**Item**	**I**	**II**	**III**	**IV**
**3**	Cirrhosis is a potential health consequence of MAFLD	0.55			

**4**	Liver cancer is a potential health consequence of MAFLD	0.71			
**7**	Abdominal pain is not a common symptom of MAFLD	0.57			
**8**	Yellow pigmentation of skin and eyes (jaundice) is not a common symptom of MAFLD	0.70			
**9**	Fatigue is not a common symptom of MAFLD	0.57			
**10**	Nausea is not a common symptom of MAFLD	0.62			
**12**	MAFLD cannot be diagnosed using blood tests	0.47			
**13**	MAFLD can be diagnosed using liver imaging	0.65			
**14**	MAFLD can be diagnosed using liver biopsy	0.62			
**15**	Obesity or being overweight is a risk factor of MAFLD	0.64			
**16**	Diabetes Mellitus is a risk factor of MAFLD	0.72			
**17**	Triglycerides is a risk factor of MAFLD	0.61			
**18**	Hypertension is a risk factor of MAFLD	0.61			
**19**	Smoking is not a risk factor of MAFLD	0.51			
**20**	Genetic predisposition is a risk factor of MAFLD	0.62			
**22**	Decreased Physical activity is a risk factor of MAFLD	0.59			
**23**	Some chronic medications may cause MAFLD	0.51			
**25**	Eating out is associated with MAFLD	0.64			
**26**	Weight loss through physical activity and diet is associated with less liver damage in patients with MAFLD	0.66			
**11**	MAFLD is not associated with any symptom		0.47		
**24**	Low fat diet can reverse liver damage in persons with MAFLD		0.56		
**27**	Increased physical activity can reverse liver damage in persons with MAFLD		0.59		
**29**	Stopping alcohol can reverse liver damage in persons with MAFLD		0.44		
**30**	MAFLD progresses very rapidly			0.60	
**31**	Persons with MAFLD have the same lifespan as people without MAFLD			0.60	
**32**	Early diagnosis and timely treatment and management reduce the disease progression			0.60	
**5**	MALFD is not communicable; it can't spread to other people				0.55
**2**	MAFLD is reversible				0.41

Notes. Metabolic dysfunction-associated fatty liver disease (MAFLD).

#### 3.2.2 Exploratory factor analysis of MAFLD-AQ

For the exploratory factor analysis of the attitudes score, all of the 17 items were extracted since none of them was over-correlated to the other (r > 0.8) or had low communalities (<0.3). The exploratory factor analysis was run over the whole sample population (n = 406) and demonstrated a three-domain structure which described 60.7 % of the variance of the 17 items. The KMO measure of sampling adequacy was found to be 0.95, with a significant Bartlett's test of sphericity (p < 0.001), indicating the adequacy of the factor analysis. The three domains of the attitudes score are summarized in Table 3.

**Table 3:** Direct Promax exploratory factor analysis of the MAFLD-AQ.

	Extraction Sums of Squared Loadings			Rotation Sums of Squared Loadings
Domain	Total	% of Variance	Cumulative %	Total
**I**	8.698	51.166	51.166	3.848
**II**	0.927	5.453	56.618	3.618
**III**	0.694	4.085	60.703	2.854

		**Domains**		
**Item No.**	**Item**	**I**	**II**	**III**
**1**	I think I should undergo medical screening for MAFLD	0.586		

**2**	I think that there is a stigma or shame associated with having MAFLD	0.634		
**3**	It makes me uncomfortable to think about MAFLD	0.758		
**4**	I am afraid of MAFLD because it is a very serious disease	0.717		
**5**	I become nervous or anxious when watching news about MAFLD	0.686		
**15**	If I developed signs or symptoms of MAFLD, I would not tell my friends or any member of my family		0.428	
**6**	I would prefer not to be friends with someone who has young onset MAFLD		0.539	
**7**	I am open to marrying someone with young onset MAFLD		0.441	
**8**	If I had MAFLD, I won 't be able to work anymore		0.758	
**9**	If I had MAFLD, I would become depressed		0.631	
**10**	If I had MAFLD, I would be worried about what people think about me and whether they will accept me		0.656	
**11**	If I had MAFLD, I would be worried about finding the best treatment			0.677
**12**	If I had MAFLD, I would be worried about the costs of medical treatment			0.717
**13**	I think that treatment for MAFLD is not worth the side effects of medications			0.482
**14**	If I had signs or symptoms of MAFLD, I might want to find answers on my own first (look online, read books, search for research articles			0.675
**16**	I am worried that doctors and hospitals do not have sufficient knowledge and expertise to handle MAFLD			0.567
**17**	If I had MAFLD, I would go to a healthcare professional and get educated on the disease			0.764

Notes. Metabolic dysfunction-associated fatty liver disease (MAFLD).

### 3.3 Content validity of knowledge and attitudes scores

#### 3.3.1 MAFLD-KQ

The internal consistency of the last version of the knowledge score was shown to be very good with a Cronbach's alpha of 0.88. The score goes from a minimum score of 0 (no knowledge) to 28 (maximum knowledge). The mean score was 15.96 ± 3.85. The four domains of the knowledge score were renamed as follows: I) Etiology, presentation and consequences/complications; II) Disease Modifying factors; III) Disease progression; and IV) Disease classification and characteristics. The means scores and the internal consistency of each sub-domain are shown in Table 4.

**Table 4:** Means scores and Internal consistency of the MAFLD-KQ domains.

Scale	Items	Mean	SD	Cronbach's alpha
		(0-28)		(n = 400)
**Total knowledge Score**		15.26	3.97	0.88
**Subdomain I** - Etiology, presentation and consequences	3, 4, 7, 8, 9, 10, 12, 13, 14, 15, 16, 17, 18, 19, 20, 22, 23, 25, 26	9.04	2.97	0.95
**Subdomain II** - Disease Modifying factors	11, 24, 27, 29	1.6	1.37	0.67
**Subdomain III** - Disease progression	30, 31, 32	3.19	2.23	0.79
**Subdomain IV -** Disease characteristics	2, 5	1.39	0.74	0.68

Notes. Metabolic dysfunction-associated fatty liver disease (MAFLD).

#### 3.3.2 MAFLD-AQ

The Cronbach's alpha of the attitudes scale was equal to 0.81 indicating a very good internal consistency. Attitudes score was divided into three subdomains which were given the following subtitles: I) Apprehension, fear, and stigma of the disease; II) Coping and living with the disease; and III) Actions towards the disease. The mean score was 42.37 ± 11.27. The minimum score is 17 and the maximum is 85. As the score increases, the more negative attitudes towards the disease are (Table 5).

**Table 5:** Means scores and Internal consistency of the MAFLD-AQ.

Attitudes Scale	Items	Mean	SD	Cronbach's alpha
		(17–85)		(n = 406)
**Total Score**		42.37	11.27	0.81
**Subdomain I –** Apprehension and stigma of the disease	1, 2, 3, 4, 5	11.67	5.64	0.89
**Subdomain II -** Coping and living with the disease	6, 7, 8, 9, 10, 15	14.28	4.58	0.61
**Subdomain III -** Actions towards the disease	11, 12, 13, 14, 16, 17	16.42	2.92	0.78

#### 3.3.3 Construct validity of MAFLD-KQ and MAFLD-AQ

**Fig. 1:**
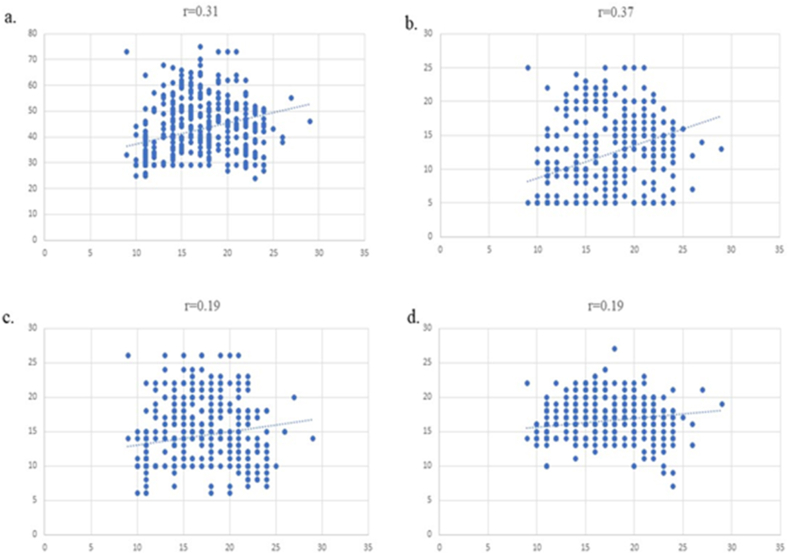
In a) Correlation between MAFLD-AQ score and MAFLD-KQ total score. In b) Correlation between MAFLD-AQ subdomain I and MAFLD-KQ total score. In c) Correlation between MAFLD-AQ subdomain II and MAFLD-KQ total score. In d) Correlation between MAFLD-AQ subdomain III and MAFLD-KQ total score *p<0.001*.

The attitudes score was positively correlated with the knowledge score (r = 0.31, p < 0.001). Correlations between each corresponding domain of MAFLD-AQ and the MAFLD-KQ total score were found to be positive [Fig. 1(a–d)].

#### 3.3.4 Known group validity of MAFLD-KQ and MAFLD-AQ

**Fig. 2:**
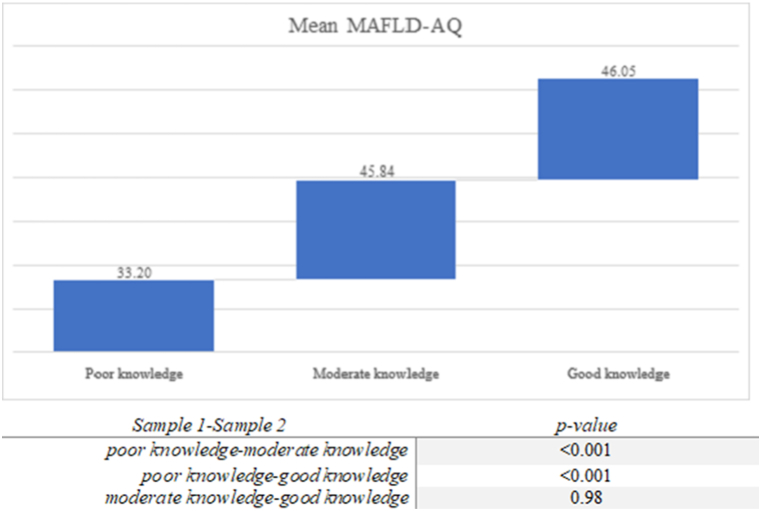
Mean Attitudes score among the three different knowledge categories.

Based on the mean ± SD of the knowledge score, it was divided into three categories. Poor knowledge was considered if a participant scored less than 12 (i.e. mean score minus the Standard Deviation (SD); moderate knowledge was considered when the total score varied between 12 and 20 (mean score plus the SD), and good knowledge regarding MAFLD disease was considered if a participant scored >20. MAFLD-AQ mean scores were then compared among the three different categories of MAFLD-KQ. As shown in Fig. 2, statistically significant differences in average attitude scores were found among *poor and moderate* knowledge as well as *poor and good* knowledge (p < 0.001). A statistically significant difference in the attitude score was not obtained between *moderate and good* MAFLD knowledge (p > 0.05)

## 4 Discussion

Since to date, there are no validated instruments available for the assessment of knowledge and attitudes regarding MAFLD among young adults in the MENA region, we developed and validated knowledge (MAFLD-KQ) and attitude (MAFLD-AQ) questionnaires. The main aim of this study was to assess the internal structure of both scales and evaluate their validity and reliability among Lebanese university students.

The stages involved in developing MAFLD questionnaires included a comprehensive content validation process. The first set of questions incorporated in the questionnaire was derived from previous research questionnaires found in the literature pertaining to MAFLD [23,40]. This first step was intended to engage the whole spectrum of information about MAFLD causation, predisposing factors, manifestations, diagnostic methods, and treatment strategies.

We first established the face validity of the questionnaires by using a preliminary survey in a pilot sample. This approach aligns with other studies on disease-specific questionnaires in which content validity is confirmed by having the questionnaire reviewed by subject-matter experts followed by conducting a pilot study [41,42].

The face validity, expressed by the acceptability and overall understanding of the questions, was reasonably good since all participants filled in the questionnaire. Subsequently, content validity that establishes whether the questions are related to one another or more constructs was assessed by using EFA. The invalidated items were removed due to either low factor loading, high inter-correlations or low communalities. Internal reliability in the final versions of the MALFD-KQ and MAFLD-AQ scores was assessed using Cronbach's alpha coefficient. Our findings indicate very good-to-excellent reliability for both scales: 0.88 for the MALFD-KQ scale and 0.81 for the MAFLD-AQ scale. These results align well with other studies validating the MAFLD knowledge and attitudes questionnaires, demonstrating similarly strong internal consistency. For instance, Du et al. (2023) concluded that the newly developed MAFLD knowledge questionnaire with items like those used in our study was a reliable and valid instrument that could be used among young Chinese adults [30]. Moreover, in a recent study conducted in 2024, Hegazi and his colleagues revealed a very good validity of the attitudes questionnaire regarding MAFLD in a sample of Egyptian adults [43]. The content validity reflected the validity of each domain of both MAFLD-KQ and MAFLD-AQ questionnaires. The presence of four subdomains in the knowledge score (disease etiology, presentation and consequences; disease-modifying factors, disease progression; and disease characteristics) displayed good factorial validity among university students. These sub-domains were also identified in previous research of MAFLD [30,44]. In his research, Du (2023) identified four out of seven factors consistent with the validation of the MAFLD knowledge questionnaire among young Chinese individuals [30]. For the MAFLD-AQ scale, EFA indicated a three-factor structure of the questionnaire that could jointly account for 60.7 % of the total observed variance. All factor loadings were above 0.3, revealing close relations between factors and items. Most items in the MAFLD-AQ questionnaire (apprehension and stigma; coping and living with the disease; actions towards the disease) were comparable to those in previous studies [45,46], but, to our knowledge, this questionnaire has not been previously validated.

The significant difference in the MAFLD-KQ scores between participants with poor vs. good knowledge demonstrated known-group validity. This validity was conducted to determine whether the MAFLD-AQ scores differed among participants with varying levels of knowledge about the disease. Our results showed that higher scores (i.e. more negative attitude) were seen among participants with moderate-to-good knowledge when compared to individuals with low MAFLD knowledge. Similar to previous studies, this result affirms that the questionnaire can detect attitudes depending on the understanding of MAFLD disease [36,38,47,48]. This finding can also be demonstrated by the positive correlation found between MAFLD-AQ total score and MAFLD-KQ total score as well as MAFLD-KQ sub-domains scores. This indicates that the higher the knowledge about MAFLD, the more negative the attitude towards it [36,44]. Although there is a correlation between the two scales, it is important to note that this correlation is relatively low (r < 0.5). This suggests variations in the attitudes scale are influenced by disease knowledge and other factors such as, medical co-morbidities, societal perceptions and personal beliefs [36,49] While our study doesn't specifically measure the prevalence of MAFLD nor details its association with different risk factors, it highlights a significant presence of MAFLD risk factors among students, many of which are modifiable, such as smoking, obesity, lack of physical activity and current co-morbidities. Consistent with recent research on the prevalence of chronic medical conditions among youth, nearly one-third of our sample are affected by one or more metabolic or cardiovascular conditions, with 32.8 % and 35 % diagnosed with DMII and Hypertension respectively [50,51]. Moreover over 50 % of participants also reported a family history of cardiovascular and metabolic diseases, including hypertension, dyslipidemia, and type 2 diabetes, emphasizing the impact of genetics alongside sociocultural habits on metabolic syndrome development. Given that MAFLD is a liver manifestation of metabolic syndrome, our results raise significant concerns about the prevalence of this often-silent disease among students.

Although our study accomplished its objective, it has some potential limitations that can help improve the outcomes in future research. One potential limitation of snowball sampling is its susceptibility to bias. As the sample expands through participant referrals, it is significantly shaped by their social networks and personal preferences. This can result in a sample that fails to accurately represent the larger population, potentially over-representing specific groups or viewpoints while neglecting others. To add, the pilot test had a relatively small sample size, and it was conducted exclusively among university students, potentially limiting its external validity. Future research may be needed to evaluate the questionnaire's appropriateness for assessing MAFLD knowledge and attitudes across the general adult population. Another possible limitation is that the study was conducted only on some university students, from five districts of Lebanon, which reduces the generalizability of the results. Furthermore, since some results were based on self-reported data, various biases, including recall bias and social desirability bias, might have been raised. In addition, the study did not assess the sensitivity of the knowledge questionnaire in responding to improvements in knowledge. This can be assessed by measuring the change in knowledge before and after an educational intervention within a randomized controlled trial.

### 4.1 Implications for clinical practice and research

Our validated MAFLD knowledge and attitudes questionnaires have important significance in clinical practice and research. To begin with, they serve as fair measurement tools for evaluating individual's current knowledge and attitudes regarding MAFLD and the efficiency of education/counselling provided by healthcare personnel [23]. In addition, in research contexts, these questionnaires help to make standardized evaluations of MAFLD-related knowledge and attitudes of different populations, to compare results, and to evaluate the effectiveness of different interventions. Moreover, the questionnaires ensure that important aspects of MAFLD are assessed including disease awareness, treatment adherence, and psychosocial factors in clinical practice and research [1]. These implications are in line with other chronic diseases where the use of validated questionnaires resulted in better patient outcomes and more efficient educational interventions [52,53].

## 5 Conclusion

In summary, MAFLD-KQ and MAFLD-AQ are reliable preliminary exploratory instruments for assessing both knowledge and attitudes of MAFLD among young adults. They offer reliable and validated tools for educational intervention planners to measure and monitor changes in knowledge and attitudes regarding MAFLD post-intervention. Future studies are still needed to evaluate their validity and reliability when used to assess attitudes and knowledge of MAFLD among other age groups. Additionally, future studies need to further confirm their dimensionality and clinical predictive value using assessment in a randomized controlled trial setting.

## CRediT authorship contribution statement

**Samah Al Tawil:** Writing – original draft, Validation, Supervision, Software, Methodology, Investigation, Formal analysis, Data curation, Conceptualization. **Mohamad Abdelkhalik:** Writing – original draft, Methodology, Investigation, Formal analysis. **Adam El Fouani:** Writing – original draft, Methodology, Investigation, Formal analysis. **Nour Allakiss:** Writing – original draft, Methodology, Investigation, Formal analysis. **Lama Mattar:** Writing – original draft, Methodology, Investigation. **Wissam H. Faour:** Writing – original draft, Supervision, Project administration, Investigation, Conceptualization. **Rajaa Chatila:** Writing – original draft, Supervision, Methodology, Investigation, Formal analysis, Conceptualization.

## Data availability statement

Data will be made available on request.

## Declaration of competing interest

The authors declare that they have no known competing financial interests or personal relationships that could have appeared to influence the work reported in this paper.

## References

[1] Chalasani N. (2018). The diagnosis and management of nonalcoholic fatty liver disease: practice guidance from the American Association for the Study of Liver Diseases. Hepatology.

[2] Younossi Z.M. (2023). The global epidemiology of nonalcoholic fatty liver disease (NAFLD) and nonalcoholic steatohepatitis (NASH): a systematic review. Hepatology.

[3] Estes C. (2018). Modeling NAFLD disease burden in China, France, Germany, Italy, Japan, Spain, United Kingdom, and United States for the period 2016-2030. J. Hepatol..

[4] Battistella S. (2023). Liver transplantation for non-alcoholic fatty liver disease: indications and post-transplant management. Clin. Mol. Hepatol..

[5] Eslam M. (2020). A new definition for metabolic dysfunction-associated fatty liver disease: an international expert consensus statement. J. Hepatol..

[6] Rinella M.E. (2023). A multisociety Delphi consensus statement on new fatty liver disease nomenclature. J. Hepatol..

[7] Kaya E., Yilmaz Y. (2024). Deciphering the implications of MAFLD and MASLD definitions in the NAFLD population: results from a single-center biopsy study. Chin Med J (Engl).

[8] Lim G.E.H. (2023). An observational data meta-analysis on the differences in prevalence and risk factors between MAFLD vs NAFLD. Clin. Gastroenterol. Hepatol..

[9] Yilmaz Y. (2023). The heated debate over NAFLD renaming: an ongoing saga. Hepatol Forum.

[10] García-Compeán D., Jiménez-Rodríguez A.R. (2022). NAFLD VS MAFLD. The evidence-based debate has come. Time to change?. Ann. Hepatol..

[11] Younossi Z. (2018). Global burden of NAFLD and NASH: trends, predictions, risk factors and prevention. Nat. Rev. Gastroenterol. Hepatol..

[12] Eslam M. (2020). The Asian Pacific Association for the Study of the Liver clinical practice guidelines for the diagnosis and management of metabolic associated fatty liver disease. Hepatol Int.

[13] Méndez-Sánchez N. (2022). Global multi-stakeholder endorsement of the MAFLD definition. Lancet Gastroenterol Hepatol.

[14] Chan K.E. (2022). Global prevalence and clinical characteristics of metabolic-associated fatty liver disease: a meta-analysis and systematic review of 10 739 607 individuals. J. Clin. Endocrinol. Metab..

[15] Yilmaz Y. (2021). The prevalence of metabolic-associated fatty liver disease in the Turkish population: a multicenter study. Hepatol Forum.

[16] Attia D. (2022). MAFLD not NAFLD is associated with impairment of health-related quality of life. J Clin Transl Hepatol.

[17] Lin S. (2020). Comparison of MAFLD and NAFLD diagnostic criteria in real world. Liver Int..

[18] Targher G. (2021). The complex link between NAFLD and type 2 diabetes mellitus - mechanisms and treatments. Nat. Rev. Gastroenterol. Hepatol..

[19] Diehl A.M., Day C. (2017). Cause, pathogenesis, and treatment of nonalcoholic steatohepatitis. N. Engl. J. Med..

[20] Younossi Z.M. (2019). The global epidemiology of NAFLD and NASH in patients with type 2 diabetes: a systematic review and meta-analysis. J. Hepatol..

[21] Alqahtani S.A. (2024). Knowledge about metabolic dysfunction-associated steatotic liver disease among the medical professionals from countries in the MENA region. Ann. Hepatol..

[22] Sibai A.M.O.O., Batal M., Adra N., El Khoury D., Hwalla N. (2010).

[23] Huang D.Q., El-Serag H.B., Loomba R. (2021). Global epidemiology of NAFLD-related HCC: trends, predictions, risk factors and prevention. Nat. Rev. Gastroenterol. Hepatol..

[24] Sayiner M. (2016). Epidemiology of nonalcoholic fatty liver disease and nonalcoholic steatohepatitis in the United States and the rest of the world. Clin. Liver Dis..

[25] Painter J.E. (2008). The use of theory in health behavior research from 2000 to 2005: a systematic review. Ann. Behav. Med..

[26] Younossi Z.M., Corey K.E., Lim J.K. (2021). AGA clinical practice update on lifestyle modification using diet and exercise to achieve weight loss in the management of nonalcoholic fatty liver disease: expert review. Gastroenterology.

[27] Chen S. (2019). Survey of nonalcoholic fatty liver disease knowledge, nutrition, and physical activity patterns among the general public in Beijing, China. Dig. Dis. Sci..

[28] Lajeunesse-Trempe F. (2023). Validation of the Fatty Liver Index for identifying non-alcoholic fatty liver disease in a Kenyan population. Trop. Med. Int. Health.

[29] Zhang W. (2019). Awareness and knowledge of nonalcoholic fatty liver disease among office employees in Beijing, China. Dig. Dis. Sci..

[30] Du Y. (2023). Development and validation of a questionnaire to assess awareness and knowledge of nonalcoholic fatty liver disease, a liver cancer etiological factor, among Chinese young adults. Asian Pac J Cancer Prev.

[31] Aucott L.S., Riddell R.E., Smith W.C.S. (2011). Attitudes of general practitioner registrars and their trainers toward obesity prevention in adults. Journal of Primary Care & Community Health.

[32] Heuckmann B., Asshoff R. (2014). German high school students' attitudes and interest in cancer and factors influencing proactive behaviour for cancer prevention. J. Cancer Educ..

[33] Lu Y. (2022). Patient attitude and determinants toward chronic diseases control: a cross-sectional survey in rural China. Front. Public Health.

[34] De Castro J., Jamali A., Ajumobi A. (2022). S1442 knowledge, attitude, and practice of internal medicine and family medicine resident physicians on non-alcoholic fatty liver disease. Official journal of the American College of Gastroenterology | ACG.

[35] Tincopa M.A. (2021). Patient disease knowledge, attitudes and behaviours related to non-alcoholic fatty liver disease: a qualitative study. BMJ Open Gastroenterol.

[36] Abdelkhalik M. (2024). Unveiling metabolic dysfunction-associated fatty liver disease: knowledge gaps and attitudes among Lebanese university students. PLoS One.

[37] Yurdugül H. (2008). Minimum sample size for cronbach's coefficient alpha: a Monte Carlo study. Eğitim fakultesi dergisi.

[38] Norman G., Cairney J. (2015).

[39] Thorndike R.M. (1995). Jum Nunnally and Ira Bernstein New York.

[40] Abdulfattah A.A. (2024). Awareness of non-alcoholic fatty liver disease and its determinants in Jazan, Saudi Arabia: a cross-sectional study. Cureus.

[41] DeVellis R.F. (2017).

[42] Rubio D.M. (2003). Objectifying content validity: conducting a content validity study in social work research. Soc. Work. Res..

[43] Hegazy M.A. (2023). Non-alcoholic fatty liver disease related knowledge among a sample of Egyptians: an exploratory cross-sectional study. Front. Public Health.

[44] Glass L. (2022). Disease knowledge, health-related quality of life, and lifestyle behavior change in patients with nonalcoholic fatty liver disease: impact of an educational intervention. Dig. Dis. Sci..

[45] Jun D.W. (2011). A study of the awareness of chronic liver diseases among Korean adults. Korean J. Hepatol..

[46] Mohamed R., Yip C., Singh S. (2023). Understanding the knowledge, awareness, and attitudes of the public towards liver diseases in Malaysia. Eur. J. Gastroenterol. Hepatol..

[47] Friedman S.L. (2018). Mechanisms of NAFLD development and therapeutic strategies. Nat Med.

[48] Polit D.F., Beck C.T. (2006). The content validity index: are you sure you know what's being reported? Critique and recommendations. Res. Nurs. Health.

[49] Gracen L. (2022). An exploration of barriers and facilitators to implementing a nonalcoholic fatty liver disease pathway for people with type 2 diabetes in primary care. Diabet. Med..

[50] Matar D. (2015). Prevalence, awareness, treatment, and control of hypertension in Lebanon. J. Clin. Hypertens..

[51] Mouhtadi B.B. (2018). Prevalence, awareness, treatment, control and risk factors associated with hypertension in Lebanese adults: a cross sectional study. Glob Cardiol Sci Pract.

[52] Anderson R.M. (1998). The third version of the diabetes attitude scale. Diabetes Care.

[53] Glasgow K.L. (1997). Parenting styles, adolescents' attributions, and educational outcomes in nine heterogeneous high schools. Child Dev..

